# Comparative Effectiveness of Mild or Conventional GnRH-Antagonist Protocols for Ovarian Stimulation in Poor Responders (Poseidon Group 4)

**DOI:** 10.3389/frph.2020.606036

**Published:** 2020-12-04

**Authors:** Hoang Le, Dong D. Nguyen, Anh T. Cao, Huong T. L. Nguyen, Dung C. Tham, Thang D. Le, Jean-Noël Hugues

**Affiliations:** ^1^IVFTA, Tam Anh General Hospital, Hanoi, Vietnam; ^2^Department of Obstetrics, Gynecology and Reproductive Medecine, Hôpitaux Universitaires Paris Seine Saint-Denis, Assistance Publique-Hôpitaux de Paris, Bondy, France; ^3^Université Paris 13, Sorbonne Paris Cité, UFR SMBH, Bobigny, France

**Keywords:** Poseidon, mild stimulation, poor responders, GnRH antagonist, IVF

## Abstract

**Background and Aims:** A panel of experts (the Poseidon Group) introduced a new and more detailed stratification for poor ovarian responders in order to predict the prognosis of IVF outcome according to the sensitivity to FSH. However, various arguments about the management strategy of these patients still remain, including the convenience and the cost. Therefore, this study was conducted to compare the efficacy of mild and conventional GnRH antagonist ovarian stimulation prescribed in patients classified in Poseidon Group 4.

**Methods:** This retrospective cohort study included 359 poor responder patients (Poseidon Group 4) treated with mild or conventional GnRH antagonist stimulation regimens from 8/2017 to 7/2019 at Tam Anh Hospital ART Center. The main outcomes were the index of Follicular Output Rate (FORT) or Follicle to Oocyte Index (FOI), the number of day-2 embryos and top-quality embryos obtained. The *t*-test and Mann–Whitney U test in SPSS v25.0 was used to analyze the continuous data and Chi-squared/Exact test was used for binary variables. Multiple linear regression analysis was done by using Stata versions 15.0 to measure association between primary endpoints with stimulation regimen controlled for covariates and possible confounding factors.

**Results:** In the overall group of poor responders, the conventional GnRH antagonist protocol performed better than the mild protocol. Subsequently, data were analyzed according to the AFC. In women with AFC < 3, no significant differences were observed between the 2 regimens regarding FORT (*p* = 0.71), FOI (*p* = 0.12), the number of day-2-embryos (*p* = 0.052) and the number of top-quality embryos (*p* = 0.26). In contrast, in women with AFC ≥ 3, mild stimulation regimen resulted in significantly poorer outcome compared to the conventional GnRH antagonist regimen, regarding FORT (*p* < 0.01), FOI (*p* < 0.01), the number of day-2-embryos (*p* < 0.01) and top-quality embryos (*p* = 0.01).

**Conclusions:** Considering poor responders classified in Poseidon Group 4, both ovarian stimulation regimens resulted in similar outcome for patients with a very low ovarian reserve (AFC < 3). In contrast, the GnRH conventional antagonist protocol with maximum initial FSH dose (300–375 IU/day) and supplementary LH (75–150 IU/day) was more effective than the mild one for patients whose ovarian reserve was less reduced. The Clinical Trial was approved by the Ethnical Biomedical Research Committee Tam Anh General Hospital.

## Background

In contemporary times, as women tend for bearing children at older age, the average age of first-time mothers is increasing in many countries (1). Thus, an increasing number of women facing a decreased ovarian reserve are asking for assisted reproductive technology to get a child (2). This subgroup represents about 37% of the overall IVF population (3), even if prognosis is very poor with a live birth rate between 6.7 and 11.4% (4–8).

Management and treatment strategies for patients displaying a poor response (POR) to ovarian stimulation are still in debate. One of the reasons is related to the heterogeneity of this subgroup of patients. Due to the lack of consensus regarding definition of POR [41 different definitions in 47 RCT (9)], some experts suggested at a Bologna meeting in 2011 a new definition based on age, ovarian reserve and previous response to stimulation (10). However, as 2 out of 3 criteria could be used to define a POR, several types of patients with different prognosis factors could be integrated within this category. To overcome those limitations of Bologna criteria, a group of scientists/clinicians published in 2016 a new classification called Poseidon (Patient–Oriented Strategies Encompassing IndividualizeD Oocyte Number) aiming at defining some subgroups with different prognosis according to their ability to get at least one euploid blastocyst for transfer (11). Besides the issue of definition, no consensual therapeutic strategy could emerge until now (12, 13). Increasing gonadotropin dose over a certain threshold is actually ineffective (14–17). A survey involving 196 ART centers in 45 nations showed that the most common protocol included GnRH antagonist regimen (53%), rFSH and hMG (43%) at a dose of 300–375 IU per day (36.7%), while 37% of centers applied mild ovarian stimulation protocol which combines Clomiphene citrate and FSH (13). AFC and AMH are efficient markers to predict both poor and hyper ovarian response but they are just “still images” and cannot reflect the dynamic of follicular growth induced by FSH. Consequently, it has been suggested to assess the actual response to FSH by measuring Follicular Output Rate (FORT) and Follicle to Oocyte Index (FOI) which both reflect the follicular sensitivity to FSH (18, 19). Improving the ovarian response to FSH is actually a key issue to enhance the reproductive prognosis of Poseidon groups 3 and 4 patients (18). Indeed, FOI improvement should increase the chance of having euploid embryos and the success rate (19).

Regarding patients classified in Poseidon group 4, an individualized treatment was recommended using GnRH antagonist protocol with the maximum starting dose of rFSH is 300 IU per day (11) alone or in association with rLH (75–150 IU per day) (20, 21). Another more cost-effective option could be the prescription of a mild stimulation. While no significant difference between mild stimulation and conventional GnRH antagonist regimen could be demonstrated regarding oocyte and embryo numbers and the clinical pregnancy rate, it might be explained by the differences in POR definition and patient selection criteria (22–24). Therefore, the objective of our study was to compare the efficacy of mild and conventional GnRH antagonist protocols in patients classified in Poseidon group 4. For that purpose, both FORT and FOI index were used to assess the ovarian response to gonadotropins.

## Materials and Methods

### Patients

This retrospective cohort study was conducted in IVFTA, Tam Anh General Hospital, from August 2017 to July 2019. All women classified as POR [group 4 of Poseidon classification: age ≥ 35 yrs, poor ovarian reserve (AFC < 5, AMH < 1.2 ng/ml)] were eligible. All patients who had alternative illness which could affect oocyte stimulation cycle, congenital uterine abnormalities, ovulatory disorders, abnormal sperm were excluded from the analysis.

### Ovarian Stimulation, Oocyte Retrieval, Zygote and Embryo Assessment, Luteal Support

All patients were prescribed one of two follicular stimulation protocols: Mild stimulation (100 mg Clomiphene Citrate from day 2 to day 7, hMG 75 IU per day from day 7) or conventional stimulation (FSH 300–375 IU per day + r.LH 75–150 IU per day from day 2).

In both protocols, GnRH antagonist was added from day 6 of ovarian stimulation. Ovulation was triggered using 10,000 IU of hCG (Pregnyl, Schering - Plough Organon, Oss, the Netherlands) when the leading follicle diameter reached 18 mm. Oocyte retrieval was performed 34–36 h after triggering ovulation. The cycle had to be canceled if there was no developing follicle or if follicular size was <15 mm after 7 days of stimulation. Embryo transfer was performed with 1 or 2 good quality cleave-stage (day 3) embryos. Transfer of more than two embryos was allowed if the woman was over 40 or if the embryo quality was not good. Supernumerary embryos were frozen and transferred in subsequent cycle.

The morphological score, the cell number, degree of fragmentation of the embryo and the uniformity of the blastomeres were assessed daily. Embryo quality were classified into grade I (no fragmentation), grade II (<20% fragmentation), grade III (20–50% fragmentation) and grade IV (>50% fragmentation). Good quality embryos included grade I and grade II embryos; Poor quality embryos included grade III and grade IV embryos. After embryo transfer, luteal support treatment was prescribed immediately until at least 12-week of pregnancy; all patients received Dydrogesterone 30 mg/day (Dusphaton® 10 mg, Abott, t.i.d. orally) and vaginal or anal suppository micro-Progesterone 600 mg/day (age < 38 yrs) or 800 mg/day (age ≥ 38 yrs) (Utrogestan® 200 mg, t.i.d. vaginal suppository or Cyclogest® 200 mg vaginal/anal suppository t.i.d. or Cyclogest® 400 mg vaginal/anal suppository b.i.d.) Measurement of serum beta hCG concentration was performed on day 12–14 post transfer and ultrasound examination to detect a gestational sac on day 21. Biochemical pregnancy (positive beta hCG in blood or urine without gestational sac at ultrasound), clinical pregnancy (presence of gestational sac including ectopic pregnancy), early pregnancy loss (miscarriage or stopped developing fetus before 12 weeks) and ongoing pregnancy (pregnancy with developed fetal heart activity till minimum 12 weeks of gestation) were all recorded.

### End Points

The primary end points of our study were the following: FORT (Follicular Output Rate: calculated as the ratio of pre-ovulatory follicle count (from 16 to 22 mm in diameter) on day of triggering/Antral follicle count under transvaginal ultrasound scans), FOI (Follicle to oocyte index: the ratio of the number of retrieved oocyte/antral follicle count), and the number of day-2 embryos, good quality embryos.

The secondary end points were the ongoing pregnancy, clinical pregnancy, early pregnancy loss, biochemical pregnancy, ovarian stimulation duration, total gonadotropin dose, ratio of the canceled cycles, ratio of fertilization.

### Statistical Analysis

SPSS (version 25.0, http://ibm-spss-statistics.com) and Stata, version 15.0 (Stata Corp, 2017, TX) for statistical analysis were used. The difference of binary outcomes was measured by using relative risk (RR) with 95% confidence interval and testing the differences was evaluated according to Fisher Exact test or Chi-square test as appropriate. For the remaining variables, the mean and standard deviation were also estimated; and compared their differences by using two-tailed *t* test or Mann – Whitney U test methods. Multiple linear regression analysis method was applied to evaluate the association between the performance of FOI, FORT, Day 2 embryos and Top-quality embryos as continuous outcomes and Mild/Conventional protocol in each group of AFC (<3 vs. ≥3) as conditional. The linear regression models were adjusted for possible covariates or confounding factors including age in year, BMI, AMH, duration of infertility in year, primary infertility. The stepwise regression of multiple variables was applied aiming at simultaneously remove the weakest correlated variables which did not significantly influence the adjusted R^2^ in the model. Level of confident was at 5%.

## Results

**Figure 1:**
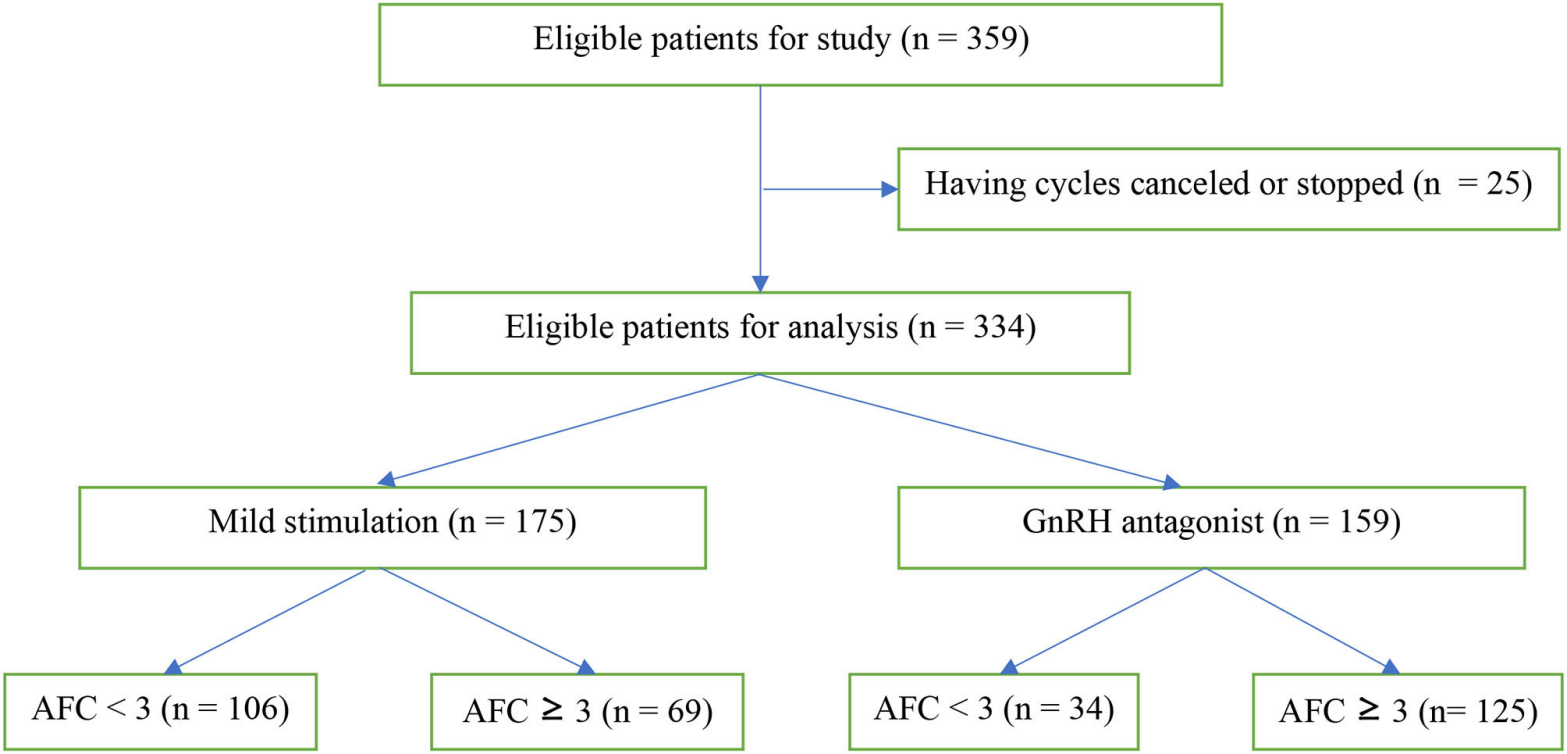
Study flow diagram.

From August 2017 to July 2019, 359 patients were eligible, 186 patients receiving the mild stimulation protocol and 173 patients receiving the conventional GnRH antagonist protocol (Figure 1). Twenty-five patients had cycles canceled or stopped at will. The remaining 334 patients were included in the analysis, consisting in 175 patients with mild stimulation and 159 patients with conventional GnRH antagonist.

### Overall Comparison of the Two Protocols (Table 1)

Baseline characteristics of patients such as age, BMI, infertility duration, rate of primary infertility, and AFC (antral follicle count) were not significantly different between the two protocols. However, basal FSH of patients treated with mild stimulation was significantly higher compared with that of patients treated with conventional stimulation (*p* < 0.01); Similarly, AMH concentration of patients that used mild ovarian stimulation was significantly lower than that of patients who used conventional stimulation.

**Table 1:** Baseline characteristics and outcome of patients treated with Mild (*n* = 175) and Conventional protocols (*n* = 159).

	**Mild (*n* = 175)**	**Conventional (*n* = 159)**	***p*-value**
Age (yrs)	42.1 ± 3.6	41.0 ± 3.5	0.46
BMI (kg/m^2^)	21.9 ± 2.0	22.1 ± 2.1	0.47
Duration of infertility (years)	4.4 ± 0.43	3.8 ± 0.41	0.52
Primary infertility *n* (%)	54 (30.9)	40 (25.2)	0.25
Basal FSH (IU/L)	12.8 ± 5.1	10.2 ± 3.2	<0.01
AMH (ng/ml)	0.3 ± 0.02	0.7 ± 0.02	<0.01
AFC (*n*)	2.3 ± 0.12	3.1 ± 0.09	0.22
FORT (%)	65 ± 4.6	75 ± 3.7	0.01
FOI (%)	60 ± 5.7	78 ± 5.2	0.01
Day 2 embryos (*n*)	0.8 ± 0.08	1.4 ± 0.12	<0.01
Top-quality embryos (*n*)	0.6 ± 0.08	0.9 ± 0.12	0.06

The overall comparison of the two regimens showed that mild ovarian stimulation was associated with a significantly lower FORT (65 ± 46 vs. 75 ± 37, *p* = 0.01), FOI (60 ± 57 vs. 78 ± 52, *p* = 0.01), number of day-2 embryo (0.8 ± 0.8 vs. 1.4 ± 1.2, *p* ≤ 0.01), as compared with conventional stimulation.

### Analysis According to the AFC

As the outcome between the two regimens was different, a subsequent analysis was performed according to the AFC in order to identify a subgroup of patients with no difference in cycle outcome. It did appear that no difference actually existed in the subgroup of patients with AFC < 3.

Table 2 shows the baseline characteristics and the cycle outcome of patients separated according to the AFC and the protocol used. In AFC < 3 group, 106 out of 140 (75.7%) patients received a mild stimulation and 34 (24.3%) a conventional one. In AFC ≥ 3 group, 69 out of 194 (35.5%) patients and 125 (64.5%) patients received, respectively, mild and conventional stimulations.

**Table 2:** Baseline characteristics and outcome of patients according the AFC analysis.

	**AFC < 3**			**AFC ≥ 3**		
	**Mild (*n* = 106)**	**Conventional (*n* = 34)**	***p*-value**	**Mild (*n* = 69)**	**Conventional (*n* = 125)**	***p*-value**
Age (yrs)	42.8 ± 3.6	41.7 ± 3.6	0.18	41 ± 3.5	40.7 ± 3.4	0.67
BMI (kg/m^2^)	21.8 ± 1.9	22.4 ± 2.4	0.28	22 ± 2.1	22 ± 2.0	0.93
Duration of infertility (years)	4.7 ± 0.46	4.2 ± 0.8	0.58	3.8 ± 0.4	3.6 ± 0.3	0.83
Primary infertility *n* (%)	35 (33)	13 (38)	0.56	19 (27.5)	27 (21.6)	0.35
Basal FSH (IU/ L)	12.3 ± 0.48	10.2 ± 0.26	0.03	13.6 ± 0.5	10.2 ± 0.4	<0.01
AMH (ng/ml)	0.3 ± 0.02	0.6 ± 0.04	<0.01	0.4 ± 0.02	0.7 ± 0.02	<0.01
AFC (*n*)	1.5 ± 0.7	1.7 ± 0.6	0.24	3.5 ± 0.5	3.5 ± 0.5	0.21
FORT (%)	79 ± 5.1	82 ± 7.2	0.71	42 ± 3.0	73 ± 3.2	<0.01
FOI (%)	74 ± 6.7	95 ± 11.2	0.12	38 ± 3.2	73 ± 4.3	<0.01
Day 2 embryos (*n*)	0.8 ± 0.08	1.1 ± 0.17	0.052	0.9 ± 0.01	1.5 ± 0.01	<0.01
Top-quality embryos (n)	0.6 ± 0.07	0.8 ± 0.13	0.26	0.6 ± 0.09	0.9 ± 0.09	0.01

Regarding primary end points, no significant difference in FORT (*p* = 0.71), FOI (*p* = 0.12), day 2 embryos (*p* = 0.052) and in the number of good embryos (*p* = 0.26) could be observed between mild and conventional stimulation in AFC < 3 group. In contrast, in AFC ≥ 3 group, mild ovarian stimulation was associated with a significantly lower FORT (42 ± 3.0 vs. 73 ± 4.3, *p* < 0.01), FOI (38 ± 3.2 vs. 73 ± 4.3, *p* < 0.01), number of day-2 embryo 2 (0.9 ± 0.01 vs. 1.5 ± 0.01; *p* < 0.01), number of good embryo (0.9 ± 0.01 vs. 1.5 ± 0.01; *p* < 0.01) as compared with conventional stimulation (Table 2).

All covariates were screened through the univariate regression model between continuous outcomes (FORT, FOI, Day 2 embryos and Top-quality embryos) with mild vs. conventional stimulation protocols. The final model of multiple linear regression included the biologically significant factors after removing the weakest factors by using backward stepwise regression approach.

The results in Table 3 showed that, in group AFC < 3, stimulation protocol (mild vs. conventional) had a significant impact on the FOI (*p* < 0.05), but not on the FORT, Day 2 embryos and Top-quality embryos (*p* > 0.05). No confounding factor was found in the multiple linear regression models. In group AFC ≥ 3, stimulation protocol (mild vs. conventional) had significant influence on all primary end points, including FOI, FORT, Day 2 embryos and Top-quality embryos (*p* < 0.05). In the modeling, only BMI was detected as a potential confounding factor to influence the relationship between stimulation protocol and Top-quality embryos (*p* < 0.05).

**Table 3:** Multiple linear regression analysis of FORT, FOI, Day 2 embryos and Top-quality embryos with mild versus conventional stimulation protocols.

**Covariate**	**AFC < 3**				**AFC ≥ 3**			
	**Co-efficient**	**SE**	***t***	***p*-value**	**Co-efficient**	**SE**	***t***	***p*-value**
**FORT as a continuous outcome**								
Stimulation protocol (mild vs. conventional)	0.01	0.11	0.06	0.949	0.26	0.06	4.47	<0.01
Age in year	−0.02	0.01	−1.44	0.15	N/A	N/A	N/A	N/A
Duration of infertility	N/A	N/A	N/A	N/A	−0.01	0.01	−1.60	0.11
Primary infertility	0.12	0.09	1.36	0.18	N/A	N/A	N/A	N/A
AMH	0.23	0.19	1.20	0.232	0.15	0.11	1.43	0.16
FSH day 2	0.026	0.01	2.64	0.09	N/A	N/A	N/A	N/A
**FOI as a continuous outcome**								
Stimulation protocol (mild vs conventional)	0.304	0.13	2.28	0.024	0.403	0.07	6.08	<0.01
FSH day 2	0.04	0.01	3.15	0.002	0.013	0.01	1.93	0.06
**Number of day 2 embryos as a continuous outcome**								
Stimulation protocol (mild vs. conventional)	0.33	0.17	1.96	0.052	0.58	0.17	3.41	0.001
Duration of infertility	N/A	N/A	N/A	N/A	−0.03	0.02	−1.33	0.18
**Number of Top-quality embryos as a continuous outcome**								
Stimulation protocol (mild vs. conventional)	0.17	0.15	1.14	0.26	0.35	0.14	2.55	0.012
BMI	N/A	N/A	N/A	N/A	0.097	0.03	3.06	0.003

*N/A, not applicable*.

As shown in Table 4, the duration of stimulation was not significantly different between the two protocols whatever the AFC < 3 (*p* = 0.39) or AFC ≥ 3 (*p* = 0.6). However, the total FSH dose required for mild ovarian stimulation was significantly lower than that required for the conventional stimulation (352 ± 13.9 vs. 3174 ± 66.5, *p* < 0.01, in AFC < 3 group; 474 ± 33.4 vs. 3261 ± 59.1, *p* < 0.01, in AFC ≥ 3 group. The fertilization rate observed with the two regimens was similar, independently of the AFC count. Regarding the cancellation rate, it was unable to measure a statistical difference between the two regimens in patients with AFC ≥ 3 due to sample size <5. Moreover, in AFC < 3 group, the cancellation rate was insignificantly different in the conventional stimulation vs. in the mild ovarian stimulation [10 (22.7%) vs. 10 (8.7%), *p* = 0.39] (Table 4).

**Table 4:** Secondary end points of two ovarian stimulation regimens.

	**AFC < 3**			**AFC ≥ 3**		
	**Mild (*n* = 106)**	**Conventional (*n* = 34)**	***p*-value**	**Mild (*n* = 69)**	**Conventional (*n* = 125)**	***p*-value**
Duration of ovarian stimulation (days)	9.8 ± 1.2	10 ± 0.8	0.39	10 ± 1.0	9.9 ± 0.7	0.6
Total FSH dose (IU)	352 ± 13.9	3,174 ± 66.5	<0.01	474 ± 33.4	3,261 ± 59.1	<0.01
Fertilized oocyte rate (%)	89 ± 3.0	77 ± 3.8	0.14	84 ± 3.5	78 ± 3.2	0.32
Cycle cancellation *n* (%)	10 (8.7)	10 (22.7)	0.39	1 (1.4)	4 (3.1)	NA

*N/A, not applicable*.

Table 5 displays the follow up for pregnancy after embryo transfer till the time of data collecting. Among 102 patients, 30 patients underwent the mild ovarian stimulation and 72 patients the conventional stimulation. There was no significant difference in pregnancy outcomes between two protocols regardless of AFC. The percentage of patients with positive β-hCG as well as the rate of biochemical pregnancy, clinical pregnancy, early pregnancy loss and ongoing pregnancy were similar between the two stimulation regimens.

**Table 5:** Cycle outcomes following the two stimulation protocols.

	**Mild (*n* = 30)**	**Antagonist (*n* = 72)**	**RR (95% CI)**
β-hCG	7 (23.3%)	24 (33.3%)	0.7 (0.34–1.45)
Biochemical pregnancy	2 (6.7%)	8 (11%)	0.6 (0.14–2.66)
Clinical pregnancy	5 (16.7%)	16 (22.2%)	0.75 (0.30–1.86)
Early pregnancy loss	1 (3.3%)	6 (8.3%)	0.4 (0.05–3.18)
Ongoing pregnancy	4 (13.3%)	10 (13.9%)	0.96 (0.33–2.82)

## Discussion

This retrospective study allows to conclude that, in Poseidon group 4 patients, both ovarian stimulation regimens results in similar outcomes when the AFC is < 3. In contrast, the GnRH conventional antagonist protocol with maximum initial FSH dose (300-375 IU/day) and supplementary LH (75–150 IU/day) is more effective than the mild **one** when AFC is ≥ 3. Our results are in good accordance with the concept that not all poor responders are the same (25).

While it has been well demonstrated that a poor ovarian response is associated with the decrease in the IVF success rate (4, 5), it is still unclear to define the optimal strategy or protocol in that situation (12, 13). Studies comparing mild stimulation protocols with conventional GnRH agonist or antagonist regimens could not demonstrate a substantial difference in terms of live birth rate but a significant improvement in the cost effectiveness (22–24).

One of the major issues clearly remains the consensus regarding the definition of the POR. Unfortunately, Bologna criteria could not fully eliminate the heterogeneity in the patients'profile and did not take into account several medical and genetic risk factors (26). Consequently, conflicting results were reported when mild and conventional stimulations were compared: some studies found no difference between the two regimens (27, 28), while, in others, the outcome of mild stimulation was lower (29–31).

Subsequently, Poseidon's stratification aimed at identifying patients according to the prognosis, women of Group 4 being characterized by a very low prognosis. In this context, to the best of our knowledge, no comparative studies have been performed until now between conventional and mild stimulations in Group 4 poor responders. Therefore, the purpose of our study was set up a comparative analysis on the effects of mild and convention stimulation in this subgroup of women. In order to assess the ovarian sensitivity to FSH, the end points were the index FORT and FOI which both reflect the dynamic response to FSH and the efficacy of the stimulation regimen (18, 19).

Due to the retrospective nature of the study, some baseline characteristics significantly differed between the two treated groups. Significant differences in the concentrations of basal FSH and AMH were observed attesting that patients in mild stimulation group tend to have a lower ovarian reserve as compared to those treated with conventional stimulation.

Interestingly, the relative efficacy of each stimulation depends on the ovarian reserve. After controlling the impact of confounding factors, our results show that, in women characterized by a low AFC (<3), the efficacy of the two regimens assessed by FORT, number of embryo day 2, and number of good quality embryos were not significantly different, except for FOI. In contrast, in women characterized by a high AFC (≥3), the efficacy of the two regimens assessed by FORT, FOI, number of embryo day 2, and number of good quality embryos significantly differed from each other. In addition, BMI was found to be the only potential confounding factor between the two regimens and the number of Top-quality embryos. This observation could be explained by the relationship between high BMI and alterations of serum metabolic parameters (increase in serum fatty acid levels) and of follicular fluid composition. As oocytes and embryos are quite sensitive to changes in their microenvironment, adverse effects of obesity on conception has been suggested (32). Furthermore, high free fatty acids levels and changes in adipokines associated with high BMI seems to affect oocyte competence (33). Finally, high BMI induces insulin resistance and hyperinsulinemia which stimulates both steroidogenesis and luteinizing hormone (LH) receptor expression in ovarian theca and granulosa cells. As a result, ovulation and oocyte maturation in obese women may be affected by the overexpression of LH (34).

Consequently, as the amount of total FSH dose was dramatically higher when using the conventional protocol, these results allow us to recommend a mild stimulation, the most cost-effective regimen in these patients with a very poor ovarian reserve. In contrast, in women with baseline AFC ≥ 3, our study shows when applying conventional protocol, the primary end points the (FORT ratio, FOI, number of embryo day 2 and number of good quality embryo) were significantly better as compared to the mild stimulation. These findings indicate that increasing the dose of FSH above a certain threshold allows to stimulate follicular recruitment, maturation and to achieve better outcomes. It has been shown that the optimal daily dose of FSH is likely to be about 300 IU and the addition of LH might improve the ovarian response to FSH, specifically in patients with moderate to severe forms of ovarian insufficiency (35). Presumably, as LH acts to stimulate theca cell androgen production, the number of FSH receptors increase at the granulosa cell levels and the sensitivity to FSH is actually improved.

In patients belonging to Poseidon group 4, the strategies currently support the use of a standard ovarian stimulation with maximum starting dose of FSH and adjuvant LH. We agree with this strategy when AFC is ≥3 because this regimen is more effective than the mild stimulation protocol. Nevertheless, when AFC is <3, the mild ovarian stimulation seems more friendly and less costly with similar outcome.

While our study was not designed to assess the pregnancy rate as a main end point, we observed that the cycle outcome was at least identical to that reported in previous studies, even though the selection criteria were very strict. The ongoing pregnancy rate was equivalent with the result of Youssef et al. (36) but remarkably higher than of Klinkert et al. (15). However, all these studies similarly concluded to the absence of difference in cycle outcome between the two protocols. In our study, biochemical pregnancy, early pregnancy loss, clinical pregnancy rates were similar in both treatment groups, in accordance with other comparable studies (27–31, 37). A recent guideline of ASRM has claimed the lack of difference in clinical pregnancy rate between the two protocols while we need further studies to conclude on other outcomes, such as cumulative pregnancy rate and live birth rate (22).

The strength of this study is the way of assessing the ovarian response to FSH by using the index FORT and FOI. This new approach allows to evaluate ovarian stimulation efficiency more accurately by reporting the number of growing follicles or oocytes to the basal AFC.

The weakness of the study is related to insufficient sample size, however the number of patients involved in this study was an overall number of patients admitted to the hospital during the study period. Besides, the study also faced to the information bias because of using retrospective data and selection bias due to no-randomized selection. The limited number of patients included in this study was insignificant to analyze the cumulative pregnancy rate and live birth rate. Furthermore, a cost effectiveness analysis should be done in further studies aimed to measure the huge difference between the total amount of FSH in mild vs. conventional stimulation, and assess the effectiveness of higher doses of gonadotrophins in the mild stimulation protocol. Because, it clearly indicated that a mild stimulation is associated with a lower consumption of FSH.Z.

## Conclusion

In women classified in Poseidon group 4, the comparative analysis of mild and conventional GnRH antagonist stimulation should be analyzed according to the baseline AFC. If the ovarian reserve is very low (AFC < 3), the efficacy between the two protocols is equivalent. Therefore, mild stimulation should be privileged, being more friendly and less costly than the GnRH antagonist protocol. In contrast, if the AFC is ≥3, the conventional GnRH antagonist protocol should be firstly considered with maximum starting dose of FSH (300–375 IU/day) and adjuvant LH (75–150 IU/day) as it seems more effective.

## Data Availability Statement

The raw data supporting the conclusions of this article will be made available by the authors, without undue reservation.

## Author Contributions

HL, DN, AC, HN, DT, and TL conserved and realized this study. J-NH reviewed the manuscript. All authors contributed to the article and approved the submitted version.

## Conflict of Interest

The authors declare that the research was conducted in the absence of any commercial or financial relationships that could be construed as a potential conflict of interest.
